# GSK3 inhibition with low dose lithium supplementation augments murine muscle fatigue resistance and specific force production

**DOI:** 10.14814/phy2.14517

**Published:** 2020-07-29

**Authors:** Kennedy C. Whitley, Sophie I. Hamstra, Ryan W. Baranowski, Colton J. F. Watson, Rebecca E. K. MacPherson, Adam J. MacNeil, Brian D. Roy, Rene Vandenboom, Val A. Fajardo

**Affiliations:** ^1^ Department of Kinesiology Brock University St. Catharines ON Canada; ^2^ Centre for Bone and Muscle Health Brock University St. Catharines ON Canada; ^3^ Department of Health Sciences Brock University St. Catharines ON Canada

**Keywords:** calcineurin, fast muscle, NFAT, PGC‐1, slow muscle

## Abstract

Calcineurin is a Ca^2+^‐dependent serine/threonine phosphatase that dephosphorylates nuclear factor of activated T cells (NFAT), allowing for NFAT entry into the nucleus. In skeletal muscle, calcineurin signaling and NFAT activation increases the expression of proliferator‐activated receptor‐gamma coactivator 1‐alpha (PGC‐1α) and slow myosin heavy chain (MHC) I ultimately promoting fatigue resistance. Glycogen synthase kinase 3 (GSK3) is a serine/threonine kinase that antagonizes calcineurin by re‐phosphorylating NFAT preventing its entry into the nucleus. Here, we tested whether GSK3 inhibition in vivo with low dose lithium chloride (LiCl) supplementation (10 mg kg^−1^ day^−1^ for 6 weeks) in male C57BL/6J mice would enhance muscle fatigue resistance in soleus and extensor digitorum longus (EDL) muscles by activating NFAT and augmenting PGC‐1α and MHC I expression. LiCl treatment inhibited GSK3 by elevating Ser9 phosphorylation in soleus (+1.8‐fold, *p* = .007) and EDL (+1.3‐fold *p* = .04) muscles. This was associated with a significant reduction in NFAT phosphorylation (−50%, *p* = .04) and a significant increase in PGC‐1α (+1.5‐fold, *p* = .05) in the soleus but not the EDL. MHC isoform analyses in the soleus also revealed a 1.2‐fold increase in MHC I (*p* = .04) with no change in MHC IIa. In turn, a significant enhancement in soleus muscle fatigue (*p* = .04), but not EDL (*p* = .26) was found with LiCl supplementation. Lastly, LiCl enhanced specific force production in both soleus (*p* < .0001) and EDL (*p* = .002) muscles. Altogether, our findings show the skleletal muscle contractile benefits of LiCl‐mediated GSK3 inhibition in mice.

## Graphical abstract

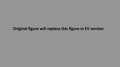

## 1 INTRODUCTION

Calcineurin is a Ca^2+^/calmodulin‐dependent serine/threonine (Ser/Thr) phosphatase that is primarily known for its role in enhancing oxidative metabolism in skeletal muscle (Ehlers, Celona, & Black, 2014; Fajardo et al., 2018; Michel, Chin, Chakkalakal, Eibl, & Jasmin, 2007; Olson & Williams, 2000; Schiaffino & Serrano, 2002). In its inactive state, an autoinhibitory domain interferes with calcineurin's catalytic domain (Rumi‐Masante et al., 2012). Under sustained elevations of intracellular [Ca^2+^]_I_, Ca^2+^/calmodulin binds to calcineurin causing displacement of the autoinhibitory domain and liberating its catalytic site for enzymatic dephosphorylation (Liu, 2009). While calcineurin has multiple substrates, its main cellular target is nuclear factor of activated T cells (NFAT) (Chakkalakal et al., 2003). Once dephosphorylated by calcineurin, nuclear localization signals on NFAT are exposed activating NFAT and allowing entry into the nucleus where it then promotes the expression of genes associated with the slow‐oxidative phenotype such as: myosin heavy chain (MHC) I, myoglobin, slow troponin, and peroxisome proliferator‐activated receptor gamma coactivator 1‐alpha (PGC‐1α) (Chakkalakal et al., 2003; Chen et al., 2015; Fajardo et al., 2018; Ryder, Bassel‐Duby, Olson, & Zierath, 2003). The promotion of these genes via calcineurin signaling and NFAT activation, particularly PGC‐1α, has shown to have implications for muscle‐based thermogenesis (Rotter et al., 2018), insulin sensitivity (Ryder et al., 2003), and fatigue resistance (Jiang, Garcia‐Roves, de Castro Barbosa, & Zierath, 2010; Sopariwala et al., 2015).

Glycogen synthase kinase 3 (GSK3) was first identified for its role in regulating glycogen synthase in muscle (Beurel, Grieco, & Jope, 2015), but is now known to phosphorylate more than 50 substrates (Beurel et al., 2015). There are two paralogous isoforms, GSK3α and GSK3β, with GSK3β being the most expressed and active isoform found within skeletal muscles (Beurel et al., 2015; McManus et al., 2005). Both isoforms are constitutively active in muscle and can be inhibited via Ser phosphorylation (Ser21 on GSK3α; and Ser9 on GSK3β), which prevents GSK3 binding to its downstream targets (Beurel et al., 2015). There are several pathways and kinases that can inhibit GSK3 via Ser phosphorylation including Akt, protein kinase A, and cyclic GMP‐dependent kinase that is activated with elevated nitric oxide (NO) (Cross, Alessi, Cohen, Andjelkovich, & Hemmings, 1995; Fang et al., 2000; Sakamoto, Arnolds, Ekberg, Thorell, & Goodyear, 2004; Sharlo et al., 2018; Zhao, Zhuang, Chen, Boss, & Pilz, 2005).

NFAT is one of many GSK3 targets; and while calcineurin acts to dephosphorylate NFAT to allow for its nuclear translocation, several studies have demonstrated that GSK3 actively re‐phosphorylates NFAT, opposing calcineurin signalling (Beals, Sheridan, Turck, Gardner, & Crabtree, 1997; Drenning et al., 2008; Martins et al., 2012; Neal & Clipstone, 2001; Sharlo et al., 2018; Sharlo, Paramonova, Turtikova, Tyganov, & Shenkman, 2019; Shen, Cseresnyes, Liu, Randall, & Schneider, 2007; Velden, Schols, Willems, Kelders, & Langen, 2008). Using a repetitive muscle contraction model, Shen et al. (2007) eloquently showed that NFAT localizes to the nucleus and is then exported back to the cytosol upon cessation of stimulation. Furthermore, GSK3 inhibition slows the export of NFAT immediately after muscle contraction thereby prolonging NFAT nuclear localization (Shen et al., 2007). Other studies in C2C12 myoblasts have shown that GSK3 inhibition increases MHC I and PGC‐1α expression, as well as myoblast fusion and differentiation, by enhancing NFAT nuclear localization and DNA‐binding (Drenning et al., 2008; Neal & Clipstone, 2001; Theeuwes et al., 2017, 2018; Theeuwes, Gosker, Schols, Langen, & Remels, 2020; Velden et al., 2008). In vivo, GSK3 inhibition has also been associated with an increase in NFAT activation and MHC I expression in rodent skeletal muscle (Martins et al., 2012; Sharlo et al., 2018, 2019). Altogether, these studies have clearly established a pathway where GSK3 inhibition activates NFAT signaling to promote MHC I and PGC‐1α expression, which could lead to an overall enhancement in muscle fatigue resistance. However, to the best of our knowledge the effects of in vivo GSK3 inhibition on muscle fatigue resistance and contractility have not yet been examined.

Lithium is an FDA approved drug that is commonly used to treat bipolar disorder and is a well‐known natural inhibitor of GSK3 (Beurel et al., 2015). Mechanistically, lithium inhibits GSK3 in two ways: (a) competing with Mg^2+^, which GSK3 requires for its kinase activity, and (b) increasing the inhibitory Ser9/Ser21 phosphorylation of GSK3α/β (Davis, Desmond, & Berk, 2018). In bipolar disorder, lithium is typically prescribed within a narrow‐window (0.5–1.0 mM serum lithium concentration) due to concerns of nephrotoxicity with chronically higher doses of lithium (Davis et al., 2018). Recent findings from our lab show that feeding mice a low dose of lithium chloride (LiCl, 10 mg kg^−1^ day^−1^) for 6 weeks in their drinking water results in a serum concentration of 0.02 mM (Hamstra et al., 2020; Kurgan, Bott, et al., 2019). Despite being well below the therapeutic range, this dose of lithium effectively inhibited GSK3 in murine cardiac and bone tissue leading to improvements in Ca^2+^ regulation and osteogenic signaling (Hamstra et al., 2020; Kurgan, Bott, et al., 2019). In the present study, we tested our hypothesis that feeding mice the same low dose of LiCl (10 mg kg^−1^ day^−1^) for 6 weeks would inhibit skeletal muscle GSK3, activate NFAT, increase PGC‐1α and MHC I expression and ultimately improve muscle fatigue resistance.

## 2 METHODS

### 2.1 Animals

Male C57Bl/6J (stock #000664, 5–6 weeks of age) were purchased from Jackson Laboratories. The mice were separated into two groups, control and LiCl fed (*n* = 16 and 17, respectively). The mice were housed in standard 12:12 hr light:dark cycles and were allowed access to standard rodent chow and water ad libitum. Lithium was fed via LiCl in drinking water in a 10 mg kg^−1^ day^−1^ dose as described previously (Hamstra et al., 2020; Kurgan, Bott, et al., 2019). After 6 weeks, all mice were sacrificed via cervical dislocation and the soleus and extensor digitorum longus (EDL) muscles were collected for contractile and western blot analyses. All animal protocols were approved by the Brock University Animal Care Committee (AUP 17‐06‐03).

### 2.2 Western blotting

A bicinchoninic acid (BCA) assay was used to determine sample protein concentration using an M2 Molecular Devices Plate Reader (Molecular Devices). Western blotting was then performed to assess phosphorylated (p)‐GSK3β (Ser9), GSK3β, p‐NFATc1, NFATc1, and PGC1‐α, MHC I, and MHC IIa. All Western blots were performed utilizing TGX BioRad PreCast 4%–15% gradient gels (#4561086: BioRad) and polyvinyldine difluoride (PVDF) membranes. All PVDF membranes were blocked with 3% (w/v) bovine serum albumin (BSA) in Tris‐buffered saline with Tween‐20 (TBST) at room temperature for 1 hr. The membranes were then incubated with primary antibodies (1:2000 dilution) overnight at 4°C. The primary antibodies for p‐GSK3β (#9336) and GSK3β (#9315) were obtained from Cell Signaling Technology. Primary antibodies for NFATc1 (#NBP2‐57739), and p‐NFATc1 (Ser172, #MAB5640) were obtained from Novus Biologicals. The primary antibody for PGC1‐α (#ST1204) was obtained from Millipore. MHC I (BA‐F8) and IIa (SC‐71) antibodies were obtained from the Developmental Studies Hybridoma Bank (University of Iowa). After incubation with the appropriate primary antibodies, PVDF membranes were then incubated at room temperature with either anti‐mouse (#7076; Cell Signaling Technology; p‐NFATc1, MHC I, MHC IIa) or anti‐rabbit (#7074; Cell Signaling Technology; PGC1‐α, NFATc1, p‐GSK3β, GSK3β) secondary antibody (1:10,000 dilution) for 1 hr. Finally, PVDF membranes were imaged after applying Millipore Immobilon Chemiluminescent HRP Substrate (#WBKLSO500, Millipore) or Clarity Max HRP substrate (BioRad) on a BioRad Chemi Doc Imager. ImageLab (BioRad) was used to quantify optical densities that were then normalized to total protein visualized on PVDF membranes with a Ponceau stain (#59803; Cell Signalling Technology).

### 2.3 Soleus and EDL contractility

For contractile analyses, intact soleus and EDL muscles were carefully dissected from the hindlimb and allowed to equilibrate in oxygenated Tyrode's solution containing 121 mM NaCl_2_, 5 mM KCl, 24 mM NaHCO_3_, 1.8 mM CaCl_2_, 0.4 mM NaH_2_PO_4_, 5.5 mM glucose, 0.1 mM EDTA, and 0.5 mM MgCl_2_, pH 7.3, that was maintained at 25°C while gassed with 95% O_2_ and 5% CO_2_. Contractile experiments were then performed as previously described (Gittings, Bunda, Stull, & Vandenboom, 2016). Briefly, using 4–0 silk sutures, soleus and EDL muscles were mounted between two platinum electrodes at their optimal length with one end tied to the arm of a dual mode servomotor (model 305B Aurora Scientific) and the other end to a fixed post. Muscles were stimulated using a biphasic stimulator (Model 701B, Aurora Scientific, Inc.) with all data sampled at 1 kHz and saved to computer for further analysis (ASI 600a software). Both EDL and soleus muscles were subjected to a force‐frequency (1–150 Hz) and fatigue protocol comprising a 70 Hz volley every 2 s for 5 min (Fajardo et al., 2016) with a sampling rate of 2000 Hz. For data analyses, peak isometric force amplitude (mN) and the maximal rates of force development (+dF/dt) and relaxation (−dF/dt) were determined during a twitch and across the range of stimulation frequencies. Peak isometric force was then normalized to muscle cross‐sectional area (CSA), which was calculated using the following formula: CSA = m/l*d*(L_f_/L_o_), where m, muscle mass (mg); l, muscle length (mm); d, mammalian skeletal muscle density (1.06 mg/mm^3^) (Mendez & Keys, 1960) and Lf/Lo is the fiber length‐to‐muscle length ratio (soleus 0.71 and EDL 0.44) (Brooks & Faulkner, 1988).

### 2.4 Statistics

All results are expressed as mean values ± *SD* of control versus lithium. Student's *t* test was used for most comparisons between the control and LiCl groups for each muscle type. For the fatigue curves, individual area‐under‐the curve values were obtained and then the averaged within their corresponding groups prior to using a Student's *t*‐test. For the force‐frequency curves, a two‐way mixed plot ANOVA was used to examine the main effects of frequency, LiCl treatment and their potential interaction. All statistical tests were conducted using GraphPad Prism 8 (GraphPad Software, Inc.) with statistical significance set to *p* ≤ .05.

## 3 RESULTS

### 3.1 Soleus and EDL weights:body weight ratios were not different between LiCl and control‐fed groups

We have previously shown that feeding mice 10 mg kg^−1^ day^−1^ of LiCl results in a serum Li concentration of 0.02 mM (Hamstra et al., 2020; Kurgan, Bott, et al., 2019). Similar to our previous studies, Table 1 shows that there were no changes in body weight between groups. There were also no significant differences between the ratios of soleus:body or EDL:body weights between the control and LiCl groups (Table 1).

**Table 1:** Body weight and soleus and EDL muscle:body weight ratio in control‐fed and LiCl‐fed mice

	Control (*n*)	LiCl (*n*)
Body weight (g)	29. 3 ± 4.5 (16)	29.6 ± 3.8 (17)
soleus (mg):body weight (g)	0.29 ± 0.04 (10)	0.30 ± 0.09 (11)
EDL (mg):body weight (g)	0.32 ± 0.06 (10)	0.36 ± 0.06 (11)

Note

A Student's *t*‐test was used to compare control versus. LiCl. All data is expressed as means ± *SD*.

### 3.2 LiCl supplementation inhibits GSK3 in both soleus and EDL muscles

**Figure 1:**
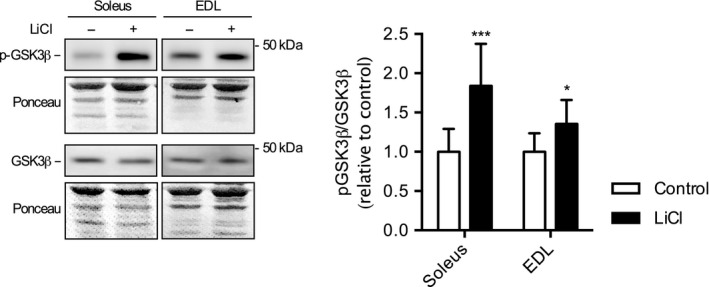
Western blot analyses shows a significant increase in glycogen synthase kinase‐3 β (GSK3β) phosphorylation (Ser9) in soleus and extensor digitorum longus (EDL) with low dose lithium (LiCl) supplementation. Phosphorylated GSK3β (p‐GSK3β) and total GSK3β were normalized to ponceau prior to calculating the ratio between p‐GSK3β/GSK3β. For p‐GSK3β and GSK3β, 7.5 μg of total protein were loaded into each well. All values are presented as relative to control. *p ≤ .05, ***p ≤ .001, using a Student's *t* test (*n* = 6 per group). All data are expressed as means ± *SD*

To provide a measure of GSK3 activation, we next measured Ser9 phosphorylation of GSK3β ‐ the dominant GSK3 isoform in murine skeletal muscle (McManus et al., 2005). Our analyses showed that Ser9 phosphorylation was significantly higher in both soleus and EDL LiCl treated muscles resulting in a greater p‐GSK3β/GSK3β ratio (*p* = .007 and *p = *.04, respectively) relative to control (Figure 1).

### 3.3 NFAT phosphorylation, PGC1‐α and MHC isoform expression

**Figure 2:**
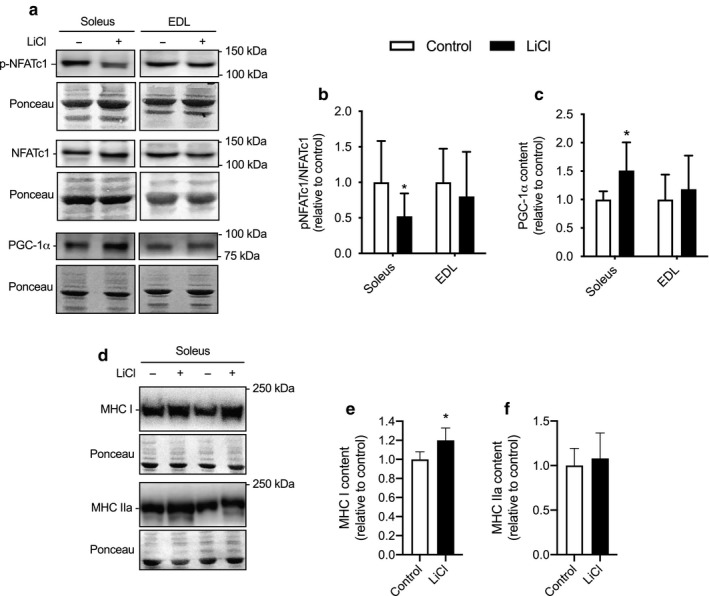
Effects of low‐dose lithium supplementation on soleus and extensor digitorum longus (EDL) NFAT activation, PGC‐1α and MHC isoform expression. (a) Representative Western blot images of phosphorylated and total nuclear factor activated T cells (p‐NFATc1 and NFATc1) and peroxisome proliferator‐activated receptor gamma coactivator 1‐alpha (PGC1‐α), all of which are downstream markers of calcineurin signaling. For pNFATc1, NFATc1, and PGC‐1α, 7.5 μg of total protein was loaded into each well. (b) Quantification of p‐NFATc1 and NFATC1 were first normalized to ponceau prior to calculating the p‐NFATc1/NFATc1 ratio. (c) Quantification of PGC1‐α content in soleus and EDL muscles in control‐fed and LiCl groups normalized to ponceau. (d) Representative Western blot images of MHC I and IIa in soleus muscles from control and lithium‐fed mice. For both isoforms, 5 μg of total protein was loaded into each well. Quantification of MHC I (e) and MHC IIa (f) normalized to ponceau. For (b,c) and (e,f), all data are expressed relative to control. *p < .05, significantly different from control, using a Student's *t* tests (*n* = 6–9 per group). All data are expressed as means ± *SD*

The effect of LiCl on NFAT activation was assessed by Western blotting for NFATc1 phosphorylation (Figure 2a,b). Though there were no significant differences in the pNFATc1/NFATc1 ratio in the EDL muscle, we did observe a 50% reduction in pNFATc1/NFATc1 in the soleus muscle of LiCl‐fed mice compared with control (*p* = .04, Figure 2b). Calcineurin signaling and NFAT activation are known to increase PGC‐1α and MHC I expression (Chen et al., 2015; Drenning et al., 2008; Martins et al., 2012; Rotter et al., 2018; Ryder et al., 2003; Sharlo et al., 2018, 2019), and corresponding well with this, our analyses showed that there was a significant increase in PGC1‐α content in the LiCl treated soleus muscles (*p* = .05), while again there was no effect of LiCl found in the EDL (Figure 2a,c). Examining MHC isoform content specifically in the soleus revealed a 1.2‐fold increase in MHC I (*p* = .04) without any change in MHC IIa (Figure 2d,e).

### 3.4 Fatigue resistance and force frequency curve analyses

**Figure 3:**
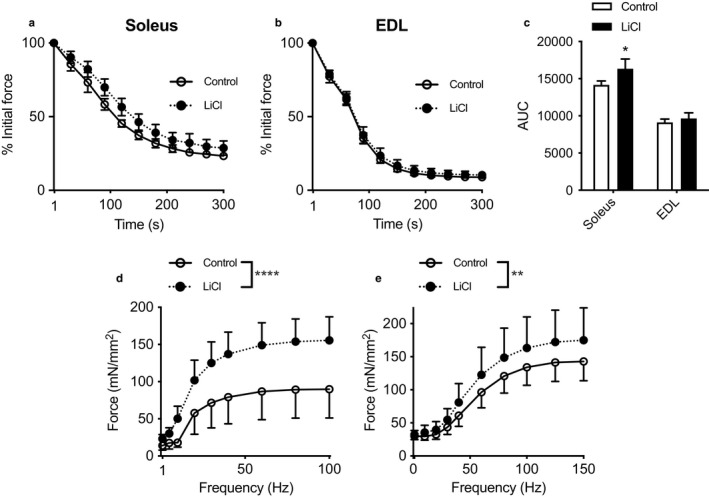
Low dose lithium supplementation (LiCl) improves soleus fatigue resistance and soleus and EDL specific force production across submaximal and maximal frequencies. (a,b) Fatigue curves and area‐under‐the‐curve (AUC, c) analyses in the soleus and EDL muscles from control‐fed and LiCl‐fed mice. (d,e) Force frequency curves in soleus and EDL muscles. For c, *p ≤ .05, significantly different from control, using a Student's *t* tests (*n* = 4–5 per group). For d,e, main effects of LiCl treatment were observed using a two‐way mixed plot ANOVA, **p ≤ .01 and **** *p* ≤ .0001 (*n* = 4–5 per group). All data are expressed as means ± *SD*

We discovered that soleus muscles collected from LiCl‐fed mice had increased fatigue resistance compared to their control‐fed counterparts (Figure 3a,c). However, there was no effect on fatigue resistance in the EDL muscles between groups (Figure 3b,c). In addition to our fatigue analyses, we also examined the effects of LiCl feeding on specific force production across submaximal and maximal stimulation frequencies. We found significant main effects of LiCl treatment on improving specific force production in both soleus and EDL muscles (Figure 3d,e). However, we found no significant differences in twitch kinetics or twitch:tetanic ratios between LiCl and control groups for both soleus and EDL muscles (Table 2).

**Table 2:** Twitch kinetics and twitch:tetanus ratio in soleus and EDL muscles from control‐fed and LiCl‐fed mice

	Control	LiCl
Soleus		
−d*F*/dt (mN/ms)	0.30 ± 0.05	0.36 ± 0.09
+d*F*/dt (mN/ms)	1.37 ± 0.26	1.78 ± 0.50
Twitch:tetanus	0.15 ± 0.05	0.15 ± 0.01
EDL		
−d*F*/dt (mN/ms)	2.13 ± 0.42	2.20 ± 0.46
+d*F*/dt (mN/ms)	4.61 ± 0.42	4.77 ± 0.82
Twitch:tetanus	0.21 ± 0.02	0.19 ± 0.02

Note

A Student's *t*‐test was used to compare control versus. LiCl (*n* = 4–5 per group). All data are expressed as means ± *SD*.

## 4 DISCUSSION

In this study, we questioned whether GSK3 inhibition with low dose LiCl treatment would enhance muscle fatigue resistance via NFAT activation and increase PGC‐1α and MHC I expression. Our results, show that GSK3β phosphorylation (Ser9) was significantly elevated in both the soleus and EDL muscles after LiCl treatment suggesting that this subtherapeutic dose of lithium (0.02 mM serum concentration) was effective in inhibiting GSK3. However, reductions in NFAT phosphorylation were only evident in the soleus from LiCl‐fed mice. Additionally, the soleus (but not EDL) from the LiCl‐fed mice displayed significant increases in downstream markers of NFAT activation, PGC‐1α and MHC I, compared to control. Consistent with this we found that soleus muscles from mice treated with LiCl displayed significantly increased fatigue resistance, whereas this was not observed in the EDL. Nonetheless, both soleus and EDL muscles from LiCl supplemented mice exhibited enhanced specific force production across sub‐maximal and maximal frequencies.

Our findings are strongly consistent with several previous studies demonstrating that GSK3 inhibition in skeletal muscle can activate NFAT and increase MHC I and PGC‐1α expression (Drenning et al., 2008; Martins et al., 2012; Sharlo et al., 2018, 2019; Theeuwes et al., 2017, 2018, 2020). In response to hindlimb unloading, the soleus muscle undergoes a slow‐to‐fast fibre type shift that is in part mediated by lowered NO content and in turn enhanced GSK3 activation (Sharlo et al., 2018, 2019). Treatment with L‐arginine (an NO donor) or a GSK3 inhibitor (AR‐A1014418) effectively lowered GSK3 activity preventing the unload‐induced decline in NFAT activation and MHC I expression in rats (Sharlo et al., 2018). In addition, subjecting unloaded rat hindlimbs to plantar mechanical stimulation also inhibits GSK3 via NO production, again preventing the unload‐induced decline in NFAT signaling and MHC I expression (Sharlo et al., 2019). In contrast to hindlimb unloading, chronic low frequency stimulation causes a fast‐to‐slow fibre type shift in rodent tibialis anterior, and Martins et al. (2012) showed that this is in part due to NO‐mediated inhibition of GSK3 and thus NFAT activation. Collectively, it is interesting to note that several studies have shown that GSK3 is inhibited with repetitive muscle activity and that its inhibition is particularly important in mediating the physiological adapations such as an increase in MHC I and PGC‐1α via NFAT signaling (Sakamoto et al., 2004; Drenning et al., 2008; Martins et al., 2012; Sharlo et al., 2019). In this respect, GSK3 inhibition could potentially act to mimic the effects of regular exercise augmenting fatigue resistance through an increase in oxidative metabolism and MHC I expression. Our results with LiCl feeding, at least in the soleus, are consistent with this notion.

The differential response between the slow‐type soleus and fast‐type EDL muscles on fatigue resistance could perhaps be explained by differences in GSK3 expression and activation. We have recently shown that when compared to the EDL muscle, the soleus has greater total GSK3 expression and activity (Kurgan, Whitley, et al., 2019). Thus, one could expect that the effects of GSK3 inhibition may be more pronounced in the soleus compared with the EDL. In addition to GSK3, slow‐type muscles also have greater calcineurin expression and activity (Chin et al., 1998; Schiaffino and Reggiani, 2011), which suggests that the relatively high GSK3 content in slow‐type muscles may act to regulate calcineurin. Owing to this, slow‐type muscles have a higher resting [Ca^2+^]_i_ compared with fast‐type muscles, and the soleus is active for longer periods throughout the day compared with the EDL (Chin et al., 1998). Thus, the soleus muscle has the necessary [Ca^2+^]_I_ and activity pattern required for calcineurin signaling and NFAT dephosphorylation. Since GSK3 “re‐phosphorylates” NFAT, inhibition of GSK3 via lithium supplementation can amplify calcineurin signaling in the soleus by activating NFAT. In contrast, in the EDL where calcineurin expression and activity are relatively low, GSK3 inhibition will have less of an effect on NFAT phosphorylation status. Therefore, future studies should determine whether activating calcineurin (i.e., with chronic low frequency stimulation, mechanical overload, or exercise) along with inhibiting GSK3 in fast muscles can lead to a synergistic effect.

Our results may be at odds with a previous study that found that lithium supplementation reduced fatigue resistance in male participants compared to those that were provided a placebo control (Tarnopolsky et al., 1996). However, the participants were only treated for 6 days and at a relatively high dose (1.0 mM serum lithium concentration) compared to our present study where mice were treated for 6 weeks at a serum concentration of 0.02 mM (Hamstra et al., 2020, Kurgan, Whitley, et al., 2019). Tarnopolsky et al. (1996) accounted their findings to a decrease in inositol triphosphate and thus impaired Ca^2+^ regulation. In contrast, we did not find any signs of altered Ca^2+^ handling or sensitivity with no changes in the rates of force development/relaxation or twitch:tetanic ratios. One potential explanation is that our study in mice utilized a much lower dose of lithium for a longer period of time allowing for the genetic changes to increase muscle fatigue resistance in the soleus (i.e., those activated by by NFAT). Even in the EDL, where we did not observe any improvements in fatigue resistance, there were no signs of muscle weakness as LiCl supplementation enhanced specific force production in both EDL and soleus. Although the underlying mechanisms explaining why GSK3 inhibition increases specific force production in the EDL and soleus is unclear, it is important to note that GSK3 is a known negative regulator of muscle mass. Specifically, GSK3 has been shown to mediate muscle atrophy through induction of E3 ligases, MuRF‐1 and Atrogin‐1 (Verhees et al., 2011). More recently, it was shown that GSK3 mediates denervation and fasting induced atrophy via desmin depolymerization and subsequent calpain‐mediated myofibril loss (Aweida et al., 2018). Conversely, GSK3 inhibition is required for IGF‐1 induced myotube hypertrophy (Rommel et al., 2001) and can stimulate myogenic differentiation and fusion (Velden et al., 2008; Kurgan et al., 2019; Velden et al., 2006). Despite the facts that we did not find any differences in soleus or EDL muscle mass with LiCl supplementation and that our measures of specific force are inherently normalized to CSA, it is possible that LiCl treatment and GSK3 inhibition may have enhanced the quality of muscle leading to improvements in muscle strength.

There is a growing body of evidence suggesting that GSK3 is a viable therapeutic target for certain neuromuscular disorders. For example, GSK3 inhibition via lithium treatment or an alternative, synthetic GSK3 inhibitor, TDZD‐8, improved muscle structure and function in a mouse model of myotonic dystrophy type 1 (Findlay et al., 2019; Jones et al., 2012). Recently, lithium treatment was also shown to improve muscle mass and strength in a mouse model of limb girdle muscular dystrophy (Findlay et al., 2019). Although the role of GSK3 in Duchenne muscular dystrophy (DMD) has not been fully investigated, elevated muscle GSK3 activity has been reported in the preclinical murine *mdx* model as well as the DMD canine model (Villa‐Moruzzi et al., 1996; Feron et al., 2009), which could be related to the decline in NO levels found in DMD muscles (Grozdanovic and Baumgarten, 1999). Interestingly, L‐arginine treatment in *mdx* mice improved muscle force production and alleviated the histopathology (Voisin et al., 2005); however, GSK3 activation was not examined. Since we show that GSK3 inhibition improves muscle fatigue resistance and specific force production in wild‐type mice, it would be of interest to specifically examine whether GSK3 inhibition could improve muscle performance and structure in the *mdx* mouse and other models of neuromuscular disease.

In summary, low dose LiCl feeding in mice inhibits GSK3 and enhances fatigue resistance in the soleus via NFAT activation and increased PGC‐1α and MHC I protein. In response to LiCl, an increase in specific force production in the soleus and EDL was found potentially due to improvements in muscle quality. Since accumulating evidence has pointed towards GSK3 as a viable target for some neuromuscular disorders, future studies should continue to investigate the therapeutic potential of low dose lithium supplementation and other GSK3 inhibitors.

## CONFLICT OF INTEREST

The authors declare that there are no conflicts of interest.

## AUTHOR CONTRIBUTIONS

KCW and VAF designed the study. KCW, SIH, RWB, CJFW, and VAF conducted the experiments. REKM, BDR, AJM, RV, and VAF contributed reagents. KCW and VAF interpreted the results and wrote the manuscript that was proofread, edited, and approved by all authors.
